# Recent Advances in Omics, Computational Models, and Advanced Screening Methods for Drug Safety and Efficacy

**DOI:** 10.3390/toxics12110822

**Published:** 2024-11-16

**Authors:** Ahrum Son, Jongham Park, Woojin Kim, Yoonki Yoon, Sangwoon Lee, Jaeho Ji, Hyunsoo Kim

**Affiliations:** 1 Department of Molecular Medicine, Scripps Research, San Diego, CA 92037, USA; ahson@scripps.edu; 2 Department of Bio-AI Convergence, Chungnam National University, 99 Daehak-ro, Yuseong-gu, Daejeon 34134, Republic of Korea; whdgka75@o.cnu.ac.kr (J.P.); woojin0681@o.cnu.ac.kr (W.K.); dbsrl0218@o.cnu.ac.kr (Y.Y.); uuni@o.cnu.ac.kr (S.L.); 3 Department of Convergent Bioscience and Informatics, Chungnam National University, 99 Daehak-ro, Yuseong-gu, Daejeon 34134, Republic of Korea; kjkh0612@o.cnu.ac.kr; 4 Protein AI Design Institute, Chungnam National University, 99 Daehak-ro, Yuseong-gu, Daejeon 34134, Republic of Korea; 5 SCICS, Prove Beyond AI, 99 Daehak-ro, Yuseong-gu, Daejeon 34134, Republic of Korea

**Keywords:** structure–activity relationship, fragment-based drug design, de novo drug design, virtual screening, quantitative structure–activity relationship

## Abstract

It is imperative to comprehend the mechanisms that underlie drug toxicity in order to enhance the efficacy and safety of novel therapeutic agents. The capacity to identify molecular pathways that contribute to drug-induced toxicity has been significantly enhanced by recent developments in omics technologies, such as transcriptomics, proteomics, and metabolomics. This has enabled the early identification of potential adverse effects. These insights are further enhanced by computational tools, including quantitative structure–activity relationship (QSAR) analyses and machine learning models, which accurately predict toxicity endpoints. Additionally, technologies such as physiologically based pharmacokinetic (PBPK) modeling and micro-physiological systems (MPS) provide more precise preclinical-to-clinical translation, thereby improving drug safety assessments. This review emphasizes the synergy between sophisticated screening technologies, in silico modeling, and omics data, emphasizing their roles in reducing late-stage drug development failures. Challenges persist in the integration of a variety of data types and the interpretation of intricate biological interactions, despite the progress that has been made. The development of standardized methodologies that further enhance predictive toxicology is contingent upon the ongoing collaboration between researchers, clinicians, and regulatory bodies. This collaboration ensures the development of therapeutic pharmaceuticals that are more effective and safer.

## 1. Introduction

Gaining insight into the mechanisms underlying pharmaceutical toxicity is an essential component of the process of creating safer therapeutic medicines. Adverse drug reactions pose substantial obstacles to the process of drug development, frequently resulting in the abandonment of drugs and increasing expenses [1]. To understand the complex causes of drug-induced toxicity, it is necessary to use a combination of modern technologies and approaches that can reveal the underlying mechanisms [2]. Advancements in omics technologies, including transcriptomics, proteomics, and metabolomics, have significantly transformed our comprehension of the molecular processes associated with drug toxicity. These technologies allow for a thorough analysis of how medications affect biological systems at a molecular level. This helps in identifying biomarkers that can detect early signs of toxicity and enhance the safety of novel therapeutic drugs [3].

The incorporation of omics data into standard toxicological assessments has fundamentally revolutionized the science of predictive toxicology. Researchers can obtain a comprehensive understanding of the molecular mechanisms behind drug toxicity by combining data from genomics, transcriptomics, proteomics, and metabolomics [3]. The utilization of multi-omics methodology not only improves our comprehension of the interactions between medication and biological systems, but also facilitates the identification of probable unintended effects and harmful byproducts. Anticipating these harmful consequences at an early stage of drug development might significantly decrease the chances of failures in later stages, therefore improving the safety and effectiveness of new medications [2].

Machine learning and in silico models have become essential tools in enhancing predictive toxicology in recent years. Quantitative structure–activity relationship (QSAR) models and multi-task deep learning algorithms have shown high levels of accuracy in predicting toxicity endpoints [4,5]. Computational models have the ability to analyze large datasets and detect patterns that are not clearly noticeable using traditional experimental methods [4]. By including omics data, these models become even more effective in making predictions, which in turn helps in identifying new biomarkers and therapeutic targets [5].

A recurring obstacle in the field of drug development is the conversion of preclinical discoveries into tangible clinical results. The introduction of micro-physiological systems (MPS) and physiologically based pharmacokinetic (PBPK) modeling has successfully tackled this issue by offering more precise forecasts of human reactions to medications [2,6]. MPS, also known as organ-on-a-chip technology, duplicates human organ systems and enables controlled investigations of the impacts of medication [7]. Pharmacokinetic models employ mathematical representations of physiological processes to predict how medications are absorbed, distributed, metabolized, and excreted in the human body [8]. When combined with omics data, these technologies provide a strong foundation for assessing the safety and effectiveness of drugs [9].

However, there are still some obstacles that continue to exist in the field of predictive toxicology [10]. The presence of diverse data types, the process of combining them, and the act of understanding their meaning provide significant challenges [11]. Due to the intricate and multi-dimensional nature of omics data, sophisticated computational approaches and tools are required for efficient analysis [3,12]. Furthermore, it is essential to perform biological validation of computational predictions in order to enhance the significance and precision of these predictions [10]. It is crucial for academics, doctors, and regulatory authorities to work together in order to provide standardized protocols and standards for using omics technology in drug development [2].

Ultimately, the integration of omics technologies with conventional toxicological assessments has yielded significant understanding of drug toxicity pathways, thereby aiding the creation of safer therapeutic agents. The use of machine learning and in silico models has greatly improved the accuracy of predicting toxicity, while the implementation of MPS and PBPK modeling has enhanced the ability to apply preclinical findings to clinical settings. Nevertheless, it is crucial to tackle the difficulties associated with data integration and interpretation in order make further progress in predictive toxicology. To fully harness the potential of these technologies for safer and more successful medication development, ongoing collaborative efforts and the establishment of standardized guidelines are essential [6].

## 2. Structure–Activity Relationships

**Figure 1:**
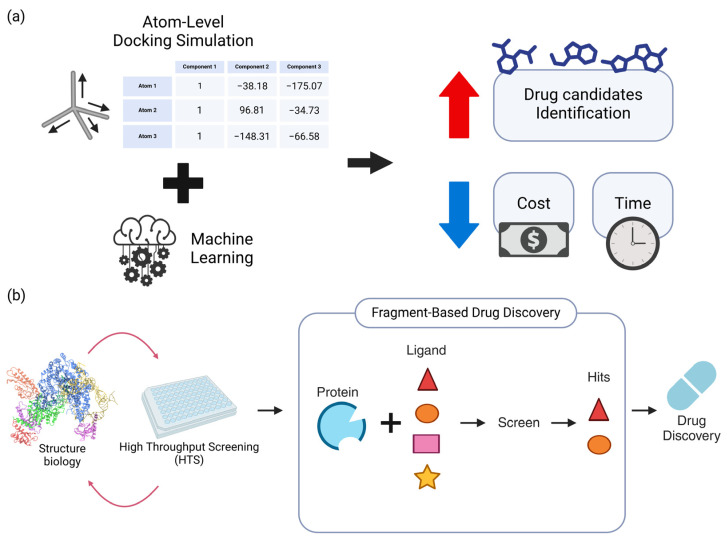
An overview of sophisticated computational drug discovery methods, including machine learning, atomic-level simulations, and fragment-based drug discovery (FBDD) candidate optimization. (**a**) A combination of atomic-level in silico docking simulations and machine learning improves drug candidate selection while reducing time and cost. The determination of the rotation and coordinates of individual atoms in the atomic-level docking data enables a more accurate and realistic simulation. This hybrid technique, combined with sophisticated machine learning algorithms, speeds up drug development by improving efficiency and accuracy. (**b**) FBDD medication-optimization scheme. Recent advances in high-throughput screening and structural biology have improved FBDD efficiency, reducing drug development time. Screening tiny molecular fragments for FBDD target protein interactions yields potential matches. These technical and methodological advances have greatly altered the drug discovery process.

Structure–activity relationship (SAR) studies are essential for comprehending the correlation between the chemical structure of a molecule and its biological activity. Recent research has utilized computational algorithms to forecast and enhance the effectiveness of potential medication candidates (Figure 1).

### 2.1. Computational SAR Models

The activity of novel chemicals has been predicted using advanced machine learning methods and molecular docking simulations. These techniques have demonstrated potential in discovering powerful inhibitors for several targets, including kinases and G-protein-coupled receptors [13,14]. Recent research has shown that the combination of machine learning algorithms with atomistic simulations can improve the accuracy of the predictions of ligand-binding free energies [15]. An example of a hybrid strategy that combines machine learning with molecular dynamics simulations has been effectively used to discover new inhibitors for SARS-CoV-2 Mpro. This demonstrates the collaborative power of computational tools and experimental validation. Furthermore, structure-based molecular modeling has played a crucial role in the analysis of SAR and the improvement of lead compounds by offering valuable information about the specific structural characteristics that contribute to biological activity and aiding in the development of more potent compounds [16,17,18]. By incorporating these computational tools into the drug development process, not only can the identification of potential therapeutic candidates be expedited, but the expense and time required for experimental testing can also be minimized [13,14] (Figure 1a).

### 2.2. Fragment-Based Drug Design

Fragment-based drug design (FBDD) is the systematic approach of discovering small chemical fragments with the capacity to bind to a certain target protein [19,20]. Afterwards, these pieces are improved to provide exceptionally potent treatment candidates. The use of high-throughput screening and structural biology methods has been crucial in the recent advancements in FBDD [21,22]. The utilization of biophysical methods, such as Nuclear Magnetic Resonance (NMR) and X-ray crystallography, has significantly enhanced the detection and enhancement of fragment hits [19,23]. Furthermore, computational techniques have been integrated into FBDD in order to augment the efficiency of fragment screening and optimization [22].

FBDD has been demonstrated as an effective method for overcoming several limitations faced in protein-targeted drug discovery, with some notable success stories in drug development [24,25]. For example: Vemurafenib (Zelboraf^®^) became, in 2011, one of the first FDA-approved FBDD-based drug-indications [26]. Using structure-guided optimization, a highly potent, BRAF V600E mutation selective inhibitor (IC50 = 31 nM) was developed based upon a weakly binding fragment (IC50 > 200 μM) [27]. By using crystal structures to strategically use functional groups, a technique which guided the process of growing an initial fragment, a drug was develped to improve melanoma survival rates in patients [28]. Sotorasib (Lumakras™), an FBDD that received FDA approval in 2021, is a prime example of FBDD tackling compounds with the opportunity to drug previously “undruggable” targets [29]. After identifying a fragment that covalently attached to KRAS G12C, the compound was further optimized by scientists via a structure-based design [30]. This led to the successful development of a drug with an objective response rate of 37.1% in non-small cell lung-cancer patients in the clinical setting, marking the first successful inhibition of KRAS in this manner [31]. One example of high selectivity from FBDD is venetoclax (Venclexta^®^), which was introduced to the market in 2016 [32]. An initial fragment screening campaign that discovered low-affinity binders to BCL-2 was followed by NMR structure-based lead optimization to deliver a potent BCL-2 selective inhibitor (Ki < 0.010 nM) with negligible off-target binding against related proteins [33]. In clinical trials, the drug has demonstrated excellent effectiveness in chronic lymphocytic leukemia, with complete response rates as high as 24% [34].

The efficacy of FBDD is further illustrated by its application in central nervous system disorders, where it has facilitated the development of medications with enhanced pharmacokinetic properties [21]. In general, FBDD is gaining momentum as a mainstream drug discovery strategy, with the potential to improve the efficiency and success rate of the development of novel therapeutic agents [35]. The combination of experimental and computational methods in FBDD is continuously progressing, offering new opportunities for the advancement of novel therapies [21,22] (Figure 1b).

## 3. Biochemical and Pharmacological Targets

**Figure 2:**
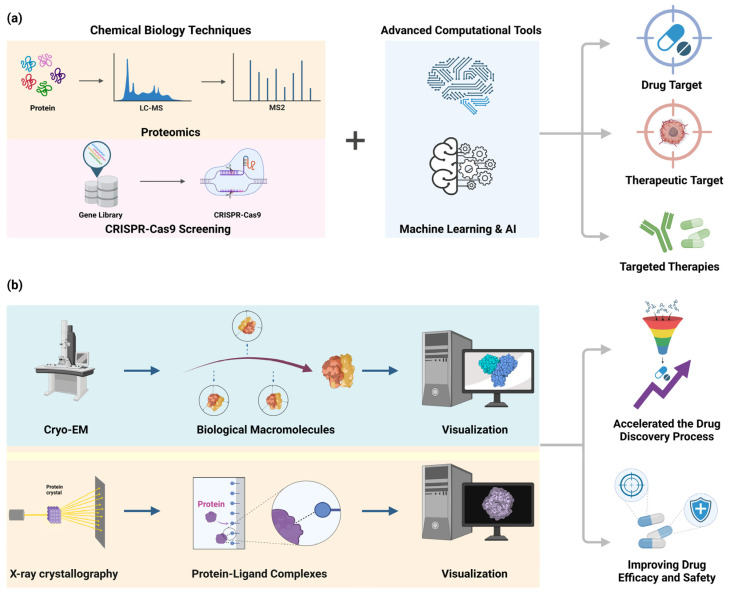
Technical methods for target identification and the understanding of mechanisms of action for the detection of biochemical and pharmacological targets. (**a**) The schematic illustrates the benefits of the integration of sophisticated computational tools, such as machine learning and AI, with proteomics and Crispr-Cas 9 screening for target identification. This has the advantage of facilitating the efficient discovery of novel therapeutic targets and the accurate identification and validation of drug targets, thereby increasing the potential for the development of targeted therapies. (**b**) The schematic demonstrates the potential to expedite the drug discovery process, and enhance the efficacy and safety of drugs, shown by the visualization of biomacromolecules and protein–ligand complexes using cryo-electron microscopy (cryo-EM) and X-ray crystallography.

Gaining knowledge about the biochemical and pharmacological targets of therapeutic action is essential for the creation of successful treatments. Current research has prioritized the identification of novel targets and the clarification of the processes by which existing medicines work (Figure 2).

### 3.1. Target Identification

Advanced techniques in molecular biology, including proteomics and CRISPR-Cas9 screening, have been instrumental in identifying novel therapeutic targets [36,37]. Proteomics-based techniques have identified new targets for cancer therapy, leading to the development of inhibitors that are both more precise and potent [37,38,39]. The application of CRISPR-Cas9 screening has enabled the precise identification and validation of medicinal targets. This method allows scientists to deactivate, activate, or modify the expression of particular genes, thus uncovering their roles in disease pathways [36,40]. The integration of these high-throughput screening approaches with advanced computational tools has also enhanced the efficiency and accuracy of target identification [37,38]. A recent study has demonstrated the advantageous use of a combination of CRISPR-based techniques and proteomics to find novel targets for therapy and acquire a deeper understanding of the mechanisms of action of small molecules [36,38,39]. These advancements have significantly accelerated the drug discovery process, providing a solid basis for the development of targeted medications [37,40] (Figure 2a).

### 3.2. Mechanism of Action

Understanding the mechanisms of action of medications requires comprehending how the medications interact with their targets on a molecular level [41]. Recent studies have utilized advanced methods such as cryo-electron microscopy (cryo-EM) and X-ray crystallography to see and understand the interactions between drugs and their targets. These techniques have allowed researchers to gain insights into how drugs bind to their targets and the structural changes that occur as a result of drug binding [42,43]. Cryo-EM offers structural insights at resolutions finer than 3 Å and can even achieve ultra-high resolutions of up to 1.2 Å. By freezing samples at extremely low temperatures, Cryo-EM allows for the observation of structures in a state close to their natural form, facilitating the identification of dynamic processes. On the other hand, X-ray analysis of protein structures is limited to the crystallized state, making it challenging to directly observe dynamic changes in living organisms. However, X-ray analysis provides high resolutions ranging from 1.5 to 2.5 Å, enabling precise structural analysis. Cryo-EM has had a significant impact in the study of complex biological macromolecules, providing almost atomic-level resolution and uncovering dynamic processes that were previously impossible to observe [44,45]. X-ray crystallography is a fundamental technique in the field of structural biology, allowing for the precise visualization of protein–ligand complexes and providing guidance for the development of drugs based on the structure of these complexes [46].

For instance, the investigation of difficult targets such as membrane proteins and time-resolved structural alterations has been facilitated by serial femtosecond crystallography (SFX) conducted with X-ray free-electron lasers (XFELs) [47,48]. The integration of X-ray crystallography with computational modeling has deepened our understanding of drug mechanisms and interactions at the atomic level [49]. This synergy has expedited the drug discovery process by establishing a comprehensive framework for the optimization of drug efficacy and safety profiles and the development of targeted therapies [50]. Recent accomplishments include the rapid development of therapeutic strategies during the COVID-19 pandemic as a result of the structural elucidation of SARS-CoV-2 proteins [51,52]. Furthermore, the efficient screening of extensive compound libraries against protein targets has been facilitated by high-throughput crystallography platforms, which have enabled fragment-based drug discovery approaches [53]. By combining structural approaches with computer modeling, we have gained a deeper knowledge of how drugs work. This has helped us build more efficient therapies [54]. The progress made in these areas has greatly expedited the process of finding new drugs, offering a strong structure for creating specific treatments and enhancing the effectiveness and safety of medications [55,56] (Figure 2b).

## 4. Drug Design and Synthetic Chemistry

**Figure 3:**
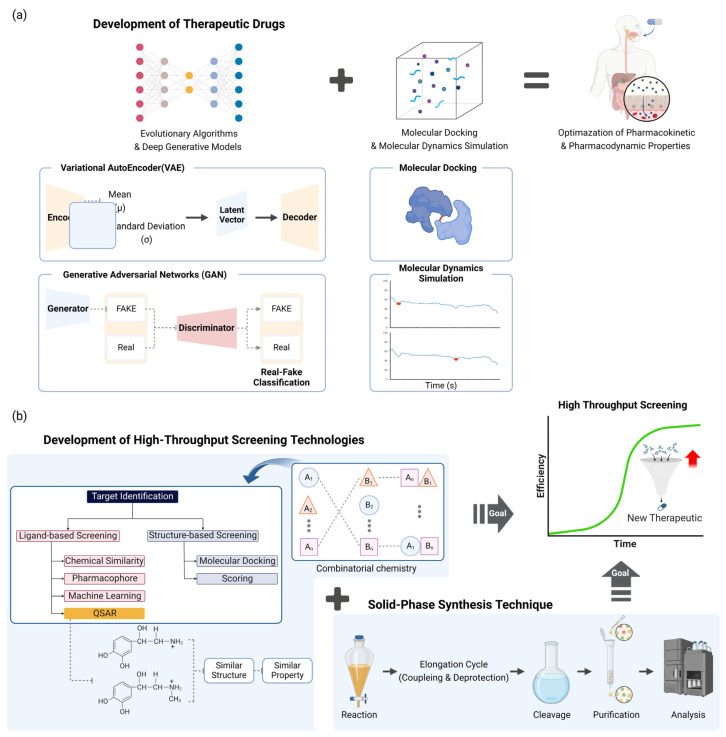
AI-based integrated strategy for synthesizing optimization and novel-drug-candidate creation and synthesis. (**a**) Deep generative models, such as VAEs and GANs, and evolutionary algorithms may identify drug candidates. This produces unique candidate drugs by using generative AI. These powerful AI-based novel drug candidate derivation methods may be used with structure-based simulation analysis tools like molecular docking or molecular dynamics simulations to find more reliable candidates. (**b**) High-throughput screening technologies, particularly combinatorial chemistry, have enhanced process efficiency while ensuring chemical diversity across large compound libraries. Quantitative structure–activity relationship (QSAR) modeling may ensure the variety of large-scale compounds, speed up assessment, and improve accuracy. Advances in solid-phase synthesis have facilitated the creation of many chemical libraries. Many medical needs may be solved by high-throughput screening and synthesizing technologies.

The design and synthesis of the novel pharmaceutical candidate are crucial stages in the drug discovery process. Recent progress in synthetic chemistry and computational approaches has expedited the creation of novel pharmaceuticals (Figure 3).

### 4.1. Automated De Novo Drug Design

Recent advancements in automated de novo drug design have facilitated the swift creation of new chemical compounds that possess specific desirable characteristics. Evolutionary algorithms and deep generative models have been used to investigate the extensive chemical space and discover potential therapeutic candidates [57]. Recent research has shown that deep generative models, such as variational autoencoders (VAEs) and generative adversarial networks (GANs), are effective in creating drug-like compounds that are both highly unique and easy to synthesize [58]. The incorporation of these models with structure-based techniques, such as molecular docking and molecular dynamics simulations, has additionally amplified the models’ ability to predict and their effectiveness [57]. Furthermore, the utilization of reinforcement learning algorithms has demonstrated potential in enhancing the pharmacokinetic and pharmacodynamic characteristics of synthesized drugs [59]. The progress made in these areas has greatly expedited the process of finding new drugs, offering a strong structure for the creation of medicines that specifically target certain conditions [57] (Figure 3a).

### 4.2. Combinatorial Synthetic Chemistry

Combinatorial chemistry is the process of quickly creating extensive collections of chemicals, which may then be tested for their biological effects [60]. Current advancements in this domain have prioritized enhancing the effectiveness and variety of compound libraries, resulting in the identification of novel lead compounds for multiple therapeutic domains. The progress in high-throughput screening methods has greatly improved the capacity to efficiently and precisely assess a huge quantity of chemicals [61]. The incorporation of computer methods, such as virtual screening and QSAR modeling, has enhanced the refinement of identifying potential candidates [50]. For example, QSAR models have been successfully applied to predict the activity of potential drug candidates against specific targets, such as the recent development of QSAR models for identifying novel inhibitors of the SARS-CoV-2 main protease [62,63]. Another notable application is the use of machine learning-based QSAR models to predict the toxicity of environmental chemicals, demonstrating the versatility of these computational approaches beyond drug discovery [64]. The synergy between combinatorial chemistry, HTS, and computational methods has not only accelerated the drug discovery process but also improved its cost-effectiveness and success rate in identifying lead compounds with desirable pharmacological properties [65].

In addition, the utilization of solid-phase synthesis techniques has simplified the creation of various chemical libraries, resulting in a more efficient and scalable procedure [66]. These advancements have not only expedited the process of discovering new drugs, but also decreased the related expenses and time required [50]. Advancements in combinatorial chemistry are expected to continue evolving and contribute to the creation of new therapies. This progress will help meet, in different disease areas, medical demands that have not been satisfied [60] (Figure 3b).

## 5. Virtual Screening

**Figure 4:**
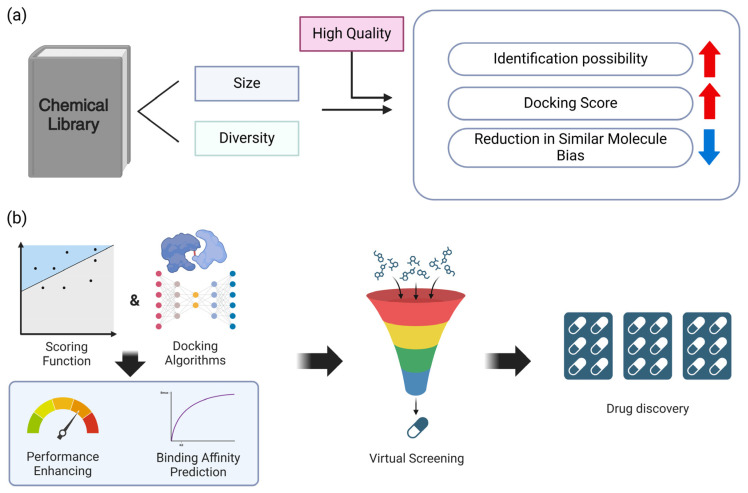
Shows the key elements to consider for virtual screening to maximize efficiency and accuracy. (**a**) Library variety and size benefits. The quantity and variety of a chemical library improve the docking score computations and candidate identification. However, if its volume grows, the library may face structural issues. Effective quality management may overcome these constraints, demonstrating that quality is as important as library quantity and variety. (**b**) A schematic showing how improved docking algorithms and scoring functions improve virtual screening efficiency and drug discovery. Docking techniques and scoring systems in virtual screening have improved performance and binding affinity prediction, improving the drug development pipeline.

Virtual screening is a computational method employed to discover new drug candidates from extensive collections of chemicals. Recent progress in virtual screening has enhanced its precision and effectiveness (Figure 4).

### 5.1. Library Size and Diversity

The magnitude and variety of chemical libraries are essential factors in determining the effectiveness of virtual screening. Recent research indicates that the presence of larger and more diversified libraries enhances the likelihood of the detection of active chemicals [67]. Nevertheless, the library’s quality is crucial, as it must encompass molecules that possess drug-like characteristics. Computational techniques such as docking have played a crucial role in expanding virtual screening libraries from millions to billions of molecules. These approaches help prioritize genuine ligands from a broad chemical space [67]. The expansion has resulted in the identification of molecules that fit better, as seen by the improvement in docking scores, which increase logarithmically with the size of the library. However, when the library size increases, there is also an increased probability of encountering artifacts that take advantage of vulnerabilities in docking scoring and sampling. Therefore, it is necessary to implement techniques to reduce the negative effects of these artifacts. Moreover, the inclination towards bio-like compounds diminishes considerably in larger libraries, thereby augmenting the investigation of novel chemical domains [68]. Hence, it is crucial to prioritize the preservation of high-quality, drug-like compounds and the effective management of artifacts in order to achieve successful virtual screening. Additionally, the inclusion of bigger and more diverse libraries can also be advantageous in this process [67,69,70] (Figure 4a).

### 5.2. Scoring Functions and Docking Algorithms

The accuracy of virtual screening has been greatly improved by advancements in scoring systems and docking algorithms. These technological breakthroughs have made it possible to identify high-affinity binders for a range of targets, including difficult ones such as protein–protein interactions. Recent research has demonstrated that combining machine learning with conventional scoring methods has enhanced performance as to many targets, resulting in more accurate predictions of binding affinities [71,72]. For instance, convolutional neural networks (CNNs) and graph neural networks (GNNs) are deep learning models that have been effectively employed to forecast protein–ligand binding affinities with a higher degree of accuracy than those achieved by conventional scoring functions [72,73]. Ensemble models that integrate multiple scoring functions have been created through the application of machine learning techniques such as gradient boosting and random forests [74]. This has led to more reliable predictions. In the context of protein–protein interactions, support vector machines (SVMs) and deep neural networks have been implemented to accurately predict binding sites and affinities, thereby surpassing the constraints of conventional docking methods [75,76]. For example, the DeepDock framework outperforms conventional docking methods by employing a combination of 3D CNNs and gradient boosting machines to predict the poses and affinities of protein–ligand binding [77]. Furthermore, reinforcement learning techniques have been implemented to iteratively optimize docking configurations and scoring functions, resulting in more precise predictions of binding modes [78]. The accuracy of virtual screening has been enhanced by these machine learning-enhanced approaches, which have also facilitated the exploration of larger chemical spaces and the identification of novel scaffolds for drug discovery [79].

In addition, the advancement of empirical scoring systems and the integration of knowledge-based methodologies have enhanced the accuracy of the prediction of binding modes and affinities [72]. Advanced sampling approaches, including shape matching, systematic search, and stochastic methods, have enhanced the precision of docking simulations [71]. These methodological improvements have increased the usefulness of virtual screening for a wider variety of targets, including those that were previously deemed challenging, such as protein–protein interactions. This is achieved by accurately simulating the intricate energy landscapes involved in these interactions [80]. As a result, the use of enhanced scoring functions and advanced docking algorithms has increased the effectiveness of virtual screening in drug discovery. This has made it easier to identify new therapeutic agents with great accuracy [81] (Figure 4b).

## 6. Computational Methods in Toxicity Prediction and Drug Safety Assessment

**Figure 5:**
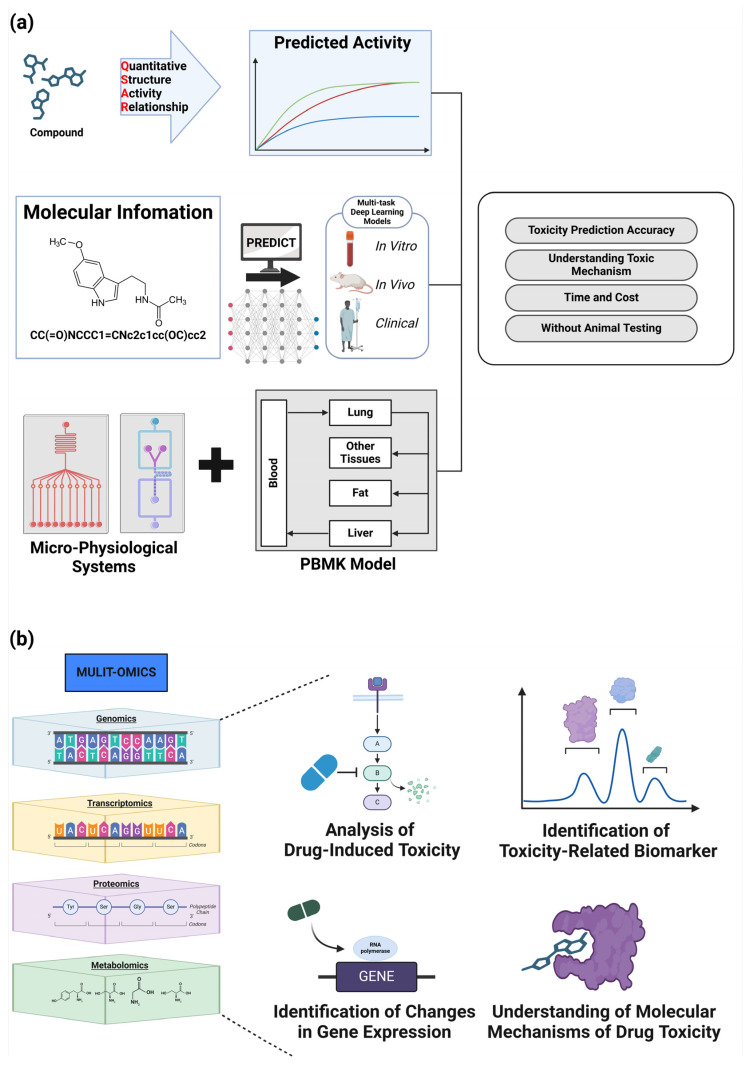
Drug safety and toxicology. (**a**) Recent advances in in silico research have enhanced the efficiency and accuracy of predictive toxicity in medication development. QSAR models estimate chemical activity and are a fast and reliable alternative to traditional experimental approaches. Multi-task deep learning models model in vitro, in vivo, and clinical toxicity data simultaneously to improve clinical toxicity prediction. Physiologically-based pharmacokinetic (PBPK) models and micro-physiological systems improve drug development efficiency and ethics. This improves human toxicity prediction without animal testing. (**b**) Multi-omics techniques are used to uncover metabolic pathway abnormalities and gene expression variations connected to toxicity. Integrating omics data with toxicological evaluations improves the understanding of drug toxicity molecular pathways. This integrated strategy helps to detect toxicity early and produce safer treatment compounds.

The application of computational methods in toxicity prediction and drug safety assessment has revolutionized drug development. These approaches enhance the efficiency of drug discovery while significantly reducing animal testing and improving toxicity prediction accuracy. This section explores computational techniques employed in toxicology, focusing on structure–activity relationships (SAR), quantitative structure–activity relationships (QSAR), and other advanced computational methods (Figure 5).

### 6.1. Predictive Toxicology

Novel chemicals’ toxicity can be predicted using computational models and in vitro testing [49,82,83]. These techniques have been employed to detect possible unintended consequences and harmful byproducts, and hence minimized the likelihood of negative outcomes in clinical studies [49,82]. Machine learning algorithms have been combined with classical in vitro assays in recent developments in predictive toxicology, resulting in the improved accuracy of toxicity predictions [49,83,84]. In silico models, such as QSAR models, offer reliable and efficient alternatives to experimental procedures [82,83]. However, it is still essential to have expert review of these predictions [84,85]. In addition, the advancement of multi-task deep learning models has made it possible to model in vitro, in vivo, and clinical toxicity data simultaneously, leading to a significant enhancement in the prediction of clinical toxicity endpoints [49]. Furthermore, the incorporation of MPS and PBPK modeling into predictive toxicology frameworks has significantly improved the capacity to forecast human toxicity without the need for animal experimentation [82,86]. These developments collectively lead to a more efficient and ethical strategy in drug development, reducing the probability of late-stage failures caused by unanticipated toxicity [2,5,49,82,87].

### 6.2. Drug Toxicity Mechanisms

Comprehending the mechanisms behind drug toxicity is crucial in the development of safer pharmaceuticals [1]. Recent research has utilized omics technologies, such as transcriptomics and metabolomics, to examine the pathways implicated in drug-induced toxicity. These observations have led to the discovery of biomarkers that can detect toxicity in its early stages. For example, transcriptomics has been used to identify changes in gene expression linked to toxic reactions, while metabolomics has revealed alterations in metabolic pathways associated with toxicity. In addition, the incorporation of these omics data with conventional toxicological evaluations has yielded a more thorough comprehension of the molecular pathways that underlie drug toxicity [1,88]. This comprehensive approach not only facilitates the early detection of potential harmful effects but also contributes to the creation of safer medical treatments [2,11].

### 6.3. Structure–Activity Relationships (SAR) in Toxicity Prediction

Developing Structure–Activity Relationship (SAR) studies determines the correlation of chemical structure with toxicodynamic effects in toxicity prediction applications; SAR analysis identifies structural features and molecular fragments that correlate with certain toxic endpoints [89,90]. Novel approaches have led to recent advances in structure–activity relationship (SAR)-based toxicity prediction. Based on established toxicological principles, fragment-based methods enable the prediction of molecular toxicity through the analysis of structural components. DEREK Nexus is an example of this type of expert system, but is based on SAR principles and used to predict toxicity via structural alerts [91]. In addition to this, read-across approaches apply SAR principles to predict the toxicity of compounds based on similarity to known chemicals, using existing toxicological data [89].

### 6.4. Quantitative Structure–Activity Relationships (QSAR) in Toxicity Modeling

QSAR models have emerged as essential components of computational toxicology, delivering quantitative toxicity predictions on the basis of molecular structure descriptors [92,93]. Machine learning algorithms such as random forests and support vector machines (SVM) have now enabled enhanced predictive performance across several toxicity endpoints [94,95]. Consensus modeling—that is, aggregating different QSAR models into a combined one—has been shown to be more robust and accurate for predicting end points like drug-induced liver injury [49,94]. Multi-task QSAR is based on the idea behind multi-task learning in machine learning or toxicology: rather than training separate single endpoint QSAR models, one can predict multiple toxicity endpoints simultaneously [49,96]. Palacios et al. demonstrated that a multi-task deep learning QSAR model achieved an average area under the curve (AUC) of 0.85 across five toxicity endpoints: hepatotoxicity, cardiotoxicity, nephrotoxicity, neurotoxicity, and genotoxicity. [49,95].

### 6.5. Physiologically Based Pharmacokinetic Model (PBPK) Modeling and Micro-Physiological Systems (MPS)

PBPK modeling and MPS come in handy when predicting drug disposition and toxicity in humans for preclinical translation, because it is difficult to extrapolate findings from animal models to the clinic [97,98]. Physiologically-based pharmacokinetic (PBPK) modeling utilizes mathematical descriptions of physiological processes to simulate how a drug is absorbed, distributed, metabolized, and excreted in the human body by combining the unique properties of the drug with its specific physiologic characteristics [95,99]. Recently, a genomic data-based approach has been implemented to address interindividual variability, disease-specific parameters have been integrated into the model, and the model has coupled with pharmacodynamic models [100]. Multi-organ-on-a-chip system/technology (MPS) technology serves as an intermediary between traditional in vitro systems and animal models. Given the necessity of replacing complex organ microenvironments, effective drug impact assessment requires highly mimetic synthesis techniques [101,102]. More recently, we have seen modelling of complex interactions between multiple organ systems, integration with patient-derived cells, and coupling to real-time sensing technologies [103]. Drug-related adverse events due to inadequate toxicity identification have been a significant cause of clinical trial failures for decades, despite regulatory oversight. The integration of PBPK models with MPS offers a robust platform for evaluating drug safety and efficacy, potentially reducing reliance on animal testing while improving the translation of preclinical data to clinical outcomes [104,105,106].

### 6.6. Computational Systems Biology in Pharmaceutical Research

Computational systems biology can now be utilized as a holistic approach to better understand the actions of a drug and predict its effects in biological systems, and it is increasingly becoming an embedded component in pharmaceutical research [107,108]. This field combines different computational techniques with large-scale biological data to understand how complex biological processes behave with respect to drug perturbations [50,109]. Network pharmacology employs systems-level analysis to identify drug targets and predict pathway-specific effects, as demonstrated by the discovery of novel anti-inflammatory compounds through target-based screening [110,111]. One especially valuable approach has been multi-scale modeling, which combines models from several biological scales—from the molecular to the organic level—to describe drug responses at the organism level [49,112]. An example of this type of modeling approach is illustrated by a multi-scale model of cardiovascular function developed to predict antihypertensive drug effectiveness and side effects [4]. In addition, in silico clinical trials have become promising tools for simulating trial results and identifying optimal values for design parameters. This ability to predict drug efficacy and toxicity has been increasingly improved by the integration of computational systems biology with the other approaches mentioned in this review (i.e., QSAR modeling and omics data analysis). This convergence accelerates our response to drug resistance while enabling more robust and safer drug development protocols, representing a paradigm shift in pharmaceutical methodology [113,114,115,116].

## 7. Challenges and Future Directions

Regulatory acceptance of computational toxicology methods has been growing, with both the FDA (U.S. Food and Drug Administration) and the EMA (European Medicines Agency) now running programs, addressing the use of these approaches, for regulatory purposes [117]. Nevertheless, these methods face challenges that preclude their standardization and overall reliability (i.e., applications across different chemical spaces). In computational toxicology, four areas of relatively recent consideration will be highlighted in the coming research: progress in predictive models for chronic and complex toxications, increased interpretability of machine learning methods-based toxicity prediction, development of multi-scale modelling approaches linking molecular events to organism-wide toxicology [98]. New updated QSARs, semi-automated and automated procedures for machine learning-enabled toxicity prediction based on modern omics data, and the integration of these predictive tools may allow rapid, actionable mechanistic insights into chemical toxicity. This approach holds real promise as a major path toward a more rational drug design process with less animal use [118,119].

Although these developments in omics technologies, computational models, and screening methods hold promise as to their potential advances in drug safety and efficacy assessments, challenges remain [120]. The integration and interpretation of these data are more challenging, as loss of statistical power can occur when integrating across multiple studies or trials [121,122]. Compelling methodological frameworks for bridging the diverse data types found in the omics studies, computational predictions, and experimental assays are necessary [4]. Despite advances in PBPK modeling and MPS, translating combined in silico and in vitro findings to clinical applications remains challenging [123]. Moreover, current methods lack predictive power as to rare and idiosyncratic drug reactions, while regulatory agencies’ acceptance of novel methodologies progresses slowly.

Several key innovations are anticipated to address these developmental challenges. In areas like explainable AI, advanced AI applications will improve the veracity and comprehension of the results of models [123]. With the advancement of multi-omics datasets, effective drugs will be predicted by integrating molecular interactions with biological systems and personalized toxicity prediction based on genome sequence profiling, along with laboratory model preparations made possible due to advancements in genomics and computational modeling [49,121]. In silico clinical trials will optimize study designs and outcome prediction methods [124], while MPS technology advancements will result in increasingly complex and physiologically relevant models, including body-on-a-chip systems used for systemic effect modeling [6,125].

## 8. Conclusions

Improvements in omics technologies, computational models, and advanced screening methods are enabling new levels of drug safety and efficacy assessment and prediction. The significant advances in transcriptomics, proteomics, and metabolomics allow for the detailed characterization of molecular mechanisms associated with drug toxicity that were previously difficult to probe, paving the way toward early detection of potential adverse effects. Multi-omics approaches feature an integrated view of drug interactions with biological systems and have made the monitoring of unwanted effects and toxic products easily detectable. Employing computational models, specifically machine learning for in silico toxicity prediction, has significantly enhanced accuracy. Multi-task deep learning algorithms and QSAR models have achieved high accuracy in predicting toxicity endpoints. The integration of omics data with these models has become a powerful approach for discovering new biomarkers and therapeutic targets. MPS and PBPK modeling have overcome the challenges associated with the translation of preclinical results to relevant clinical outcomes by providing more predictive data points in regard to human responses to drugs.

Even though these scientific advancements have been made, integrating and interpreting such biological data remains difficult, given the high complexity and multi-dimensionality of omics data. Validation of computational predictions with biological information will continue to be an essential step toward their relevance and validity. There is an urgent need for a continued dialogue between researchers, clinicians, and regulatory authorities to develop a consensus regarding standardized approaches for omics technology application in drug development. Recent advances in SAR studies, particularly with regard to computational SAR models, have increased our capacity to predict and optimize the efficiency of therapeutic drugs. Recent technological advances, such as CRISPR-Cas9 and cryo-electron microscopy, have improved the understanding of biochemical and pharmacological targets, aiding in the development of more targeted therapeutics. Automated de novo drug design coupled with combinatorial chemistry has accelerated compound generation, while advanced virtual screening methodologies have enhanced candidate identification through improved library design, scoring functions, and docking algorithms. 

The combination of predictive toxicology and mechanism-based studies has enhanced the safety profiling of new drugs, allowing for faster and more ethical drug development processes. Together, these advances illustrate how drug discovery is benefiting from the application of integrated computational and experimental approaches for both small molecule and therapeutic antibody discovery, leading to better-tolerated and more effective therapies. Collectively, this synergistic strategy, along with continual developments in predictive toxicology, will certainly be essential for producing the most secure, and also dependable, restorative agents for patients while minimizing the expense of late-stage drug-development failings.

## Data Availability

No new data were created in this study.
